# The Use of Point-of-Decision Prompts to Increase Stair Climbing in Singapore

**DOI:** 10.3390/ijerph10010210

**Published:** 2013-01-07

**Authors:** Robert Alan Sloan, Benjamin Adam Haaland, Carol Leung, Falk Müller-Riemenschneider

**Affiliations:** 1 Physical Activity Center of Excellence, Health Promotion Board, 3 Second Hospital Ave, S168937, Singapore; E-Mail: Carol_Leung@HPB.gov.sg; 2 Center for Quantitative Medicine, Office of Clinical Science, Duke-NUS Graduate Medical School, 8 College Road, S169857, Singapore; E-Mail: benjamin.haaland@duke-nus.edu.sg; 3 Department of Statistics and Applied Probability, National University of Singapore, S169857, Singapore; 4 Saw Swee Hock School of Public Health, National University of Singapore, 16 Medical Drive, S117597, Singapore; E-Mail: Falk.mueller-riemenschneider@nuhs.edu.sg; 5 Institute for Social Medicine, Epidemiology and Health Economics–Charité University Medical Center Berlin, 10117, Berlin, Germany

**Keywords:** point-of-decision prompts, physical activity, health promotion, stairs, Asia

## Abstract

Physical inactivity is a significant contributor to worldwide mortality and morbidity associated with non-communicable diseases. An excellent avenue to incorporate lifestyle physical activity into regular routine is to encourage the use of stairs during daily commutes. We evaluated the effectiveness of point-of-decision prompts (PODPs) in promoting the use of stairs instead of the escalators in a Singapore Mass Rapid Transit (MRT) station. We measured the number of stair climbers before the PODPs were put up, during the 4 weeks that they were in use, and 2 weeks after they were removed. Measurements at a no intervention control site were additionally taken. The use of stair-riser banners was associated with an increase in the number of people using the stairs by a factor of 1.49 (95% CI 1.34–1.64). After the banners were removed, the number of stair climbers at the experimental station dropped to slightly below baseline levels. The Singapore MRT serves a diverse multi-ethnic population with an average daily ridership of over 2 million and 88 stations island-wide. An increase of physical activity among these MRT commuters would have a large impact at the population level. Our findings can be translated into part of the national strategy to encourage an active lifestyle in Singaporeans.

## 1. Introduction

The World Health Organization reports that approximately 65% of all worldwide deaths are associated with non-communicable diseases (NCDs) and the rate is expected to climb another 15% in less than 20 years [1]. Concomitantly, Singapore precipitously went from a developing country to a modern developed nation in one generation and has not proven immune to the worldwide increases in NCDs that other industrialized countries are facing. According to the Singapore Ministry of Health [2] the incidence of obesity and type II diabetes have been increasing at a steady rate since 1998. Human capital and productivity are two of Singapore’s key resources; therefore population health strategies that aim to prevent or delay NCDs are imperative. More sophisticated and sustainable public health approaches to increasing physical activity and reducing sedentary behavior are needed in order to help reduce the fiscal and social costs associated with poor health related quality of life, non-communicable diseases, and premature mortality. 

Concepts such as active living [3] and zero point thinking, where some activity is better than none [4], together provide the underpinning that transitory and well-spaced increments of physical activity can mitigate incidence of NCDs and likely improve adherence to higher volumes of activity [4]. The Singapore Mass Rapid Transit (MRT) system serves a diverse multi-ethnic population, composed of approximately 75% Chinese, 15% Malays, 8% Indians, and 2% others, and has an average daily ridership of over 2 million, with 88 stations across the country [5] thus providing a potential strategic environment to induce more physical activity and increase energy expenditure. A valid tactic to increase population physical activity and energy expenditure is to encourage use of stairs during daily commutes [6,7]. Physiologically, stair-climbing is considered a moderate to vigorous form of intermittent physical activity [8] associated with improvements in cardiovascular fitness, quadriceps strength, bone mineral density, lipid profiles, and stroke incidence [9,10,11,12]. 

Many studies have examined use of point-of-decision prompts (PODP) such as signs, posters or banners to encourage individuals to use stairs instead of elevators or escalators in various built environment settings [13,14,15,16]. The findings of such studies are promising while effectiveness appears to be context-dependent. For example, motivational posters significantly increased stair use in women but not men in Berlin underground stations [14]. In a study done in Japan, stair-risers PODPs increased stair use in both genders, and the effect was especially dramatic in students [15]. However, similar interventions had no effect in the Chinese population in Hong Kong [16]. 

**Figure 1:**
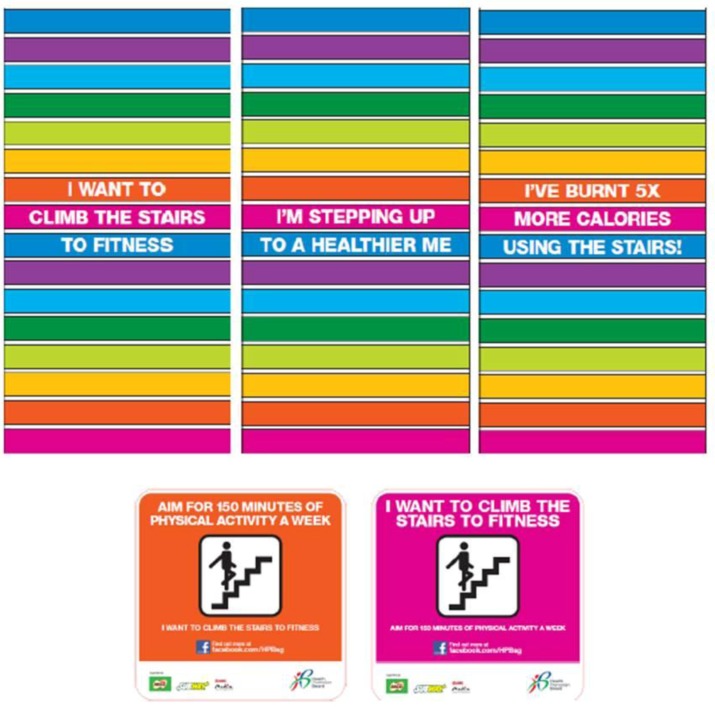
Design of point-of-decision prompts (PODPs) used. Stair-riser banners and floor stickers bearing physical activity messages were used as PODPs to promote the use of stairs instead of the adjacent escalator.

We evaluated the effectiveness of colorful stair-riser PODPs (Figure 1) in an underground station by monitoring the number of stair climbers before, during and after the implementation of prompts. A recent systematic review found some evidence that stair-riser banners might perform better than wall posters [6]. Only one other study [15] has investigated the effectiveness of stair-riser PODPs in a train station but the investigators also used wall posters simultaneously making it difficult to discern effectiveness of stair-risers alone. This study is unique in that it was conducted in Singapore and it also accounted for a control station in its design. The aim of this investigation was to determine the effectiveness of stair-riser PODPs on incidence of stair climbing in Singapore.

## 2. Methods

Two centrally located underground air-conditioned MRT stations were chosen for this study. Both stations were along the same train line and were not interchange stations. Both the stations have a 30-step staircase of identical dimensions (16 cm × 328 cm) located adjacent to an escalator. In the experimental station, a large floor sticker at the base of the stairs and colorful stair-riser PODPs bearing motivational physical activity messages “I want to climb the stairs for fitness” and “I’ve burnt 5× more calories using the stairs!” were used to encourage stair climbing (Figure 1). In the control station, no PODPs were used. 

Using a standard hand-held push button counter, three staff were trained by the Health Promotion Board to identify and count males, females and trains respectively. The trained counters were positioned at the bottom of the staircase with a clear line of sight. Counters were given permission from SMRT to do the stair climb count but not to ask questions or interact with commuters. The number of male and female stair climbers and number of trains (Table 1) were counted over 3 time of day periods (07:45–08:45 h, 12:00–13:00 h, 19:45–20:45 h) replicated on two separate weekdays (T&TH) generating a total of six observations for each station. The counts were taken during the last week of each observation period (pre = weeks 1–2, intervention = weeks 3–6, post = weeks 7–8). The observation counts occurred during a non-holiday period from August to November 2011. The climate in Singapore was consistent throughout the observation period with minimal fluctuations in mean daily temperature (27.1–27.9 °C) [17]. 

**Table 1:** Number of stair climbers and trains in the experimental and control stations at different periods.

Period	Experimental Station				Control Station			
	Male	Female	Total	Number of trains	Male	Female	Total	Number of trains
**Pre**	666	575	1,241	257	290	216	506	239
**Intervention**	1,115	1,164	2,279	247	243	216	459	246
**Post**	585	555	1,140	254	254	199	453	253

Although stairway dimensions and design of the control and experimental stations were identical, the stations themselves were different; therefore the variance in stair climbing rates between the two stations could have occurred because of intervention or station to station differences. To better account for possible confounding differences in station design as well as other confounders (gender, time of day, and number of trains) a negative-binomial generalized estimating equation (GEE) model was used. The mean number of stair climbers during the PODP intervention was compared to the mean number of stair climbers while the PODP messages were not present in the context of this model. To assess the independent impact of each variable, a multivariate negative binomial GEE model was constructed using one-variable-at-a-time backwards-stepwise variable selection with a *p*-value ≥0.05 exit criterion. Associations among observations within experimental and control stations were accounted for with robust sandwich standard error estimates [18]. To assess the impact of variables independently associated with number of people taking the stairs on the effects of one another, two-way interactions were incorporated into the multivariate model, once again using one-variable-at-a-time backwards stepwise variable selection with a *p*-value ≥0.05 exit criterion. *p*-values <0.05 were considered statistically significant. Analysis was performed using SAS version 9.2 and R 2.11.1. Due to its observational nature, this study was exempted by the Health Promotion Board Medical and Dental Ethics Committee.

## 3. Results

**Figure 2:**
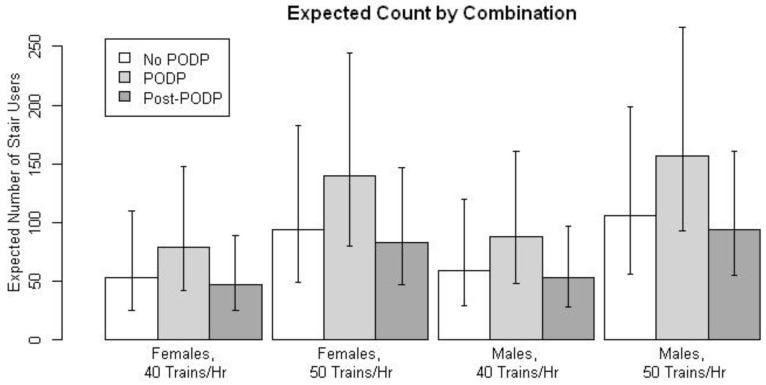
Expected number of stair climbers by gender, number of trains, PODP intervention, and post-PODP. Note that 40 trains/h and 50 trains/h are approximately the observed first and second tertiles, respectively.

Table 1 provides a descriptive representation of stair climbers, gender, and number of trains by PODP period and station. Table 2 provides univariate and multivariate results of the GEE model. All variables except evening rush hour, as compared to morning rush hour, were associated with differences in the mean number of people taking the stairs. On average, more people used the stairs of male gender, during PODP intervention, and during periods with more trains. On the other hand, less people used the stairs on average during lunchtime and after removal of PODP intervention, relative to before intervention. One-at-a-time backwards removal of variables having *p*-values ≥0.05 resulted in a multivariate model containing the variables gender, PODP intervention, post-PODP, and number of trains. After accounting for the effects of gender, PODP intervention, post-PODP, and number of trains, neither lunchtime nor evening rush hour was found to differ from morning rush hour. It is of note that number of trains and lunchtime *vs.* evening or morning rush hour are both proxies for number of people moving through the station and largely collinear and therefore not readily distinguishable in this setting. The independent associations of gender, PODP intervention, post-PODP, and number of trains with mean number of people taking the stairs were qualitatively similar to the individual associations. Expected number of stair climbers by gender, number of trains, PODP intervention, and post-PODP messages are summarized in the Figure 2. 

**Table 2:** Univariate and multivariate results of the Generalized Estimating Equation (GEE) model. The incidence ratio and the respective 95% confidence interval are shown.

	Univariate			Multivariate ^†^		
Variable	Incidence Ratio	95% C.I.	*p*-value	Incidence Ratio	95% C.I.	*p*-value
Male Gender *^a^*	1.14	(1.07, 1.22)	<0.001	1.12	(1.09, 1.15)	<0.001
PODP intervention *^b^*	1.34	(1.25, 1.44)	<0.001	1.49	(1.34, 1.64)	<0.001
Post-PODP *^b^*	0.86	(0.80, 0.92)	<0.001	0.88	(0.81, 0.97)	0.007
Lunch-time *^c^*	0.40	(0.21, 0.77)	0.006	1.16	(0.28, 4.75)	0.83
Evening rush hour *^c^*	1.50	(0.54, 4.19)	0.44	1.10	(0.39, 3.04)	0.86
Number of Trains	1.06	(1.05, 1.07)	<0.001	1.06	(1.05, 1.07)	<0.001

*^a^* female gender is reference; *^b^* no PODP is reference; *^c^* morning rush hour is reference; ^†^ multivariate model contains gender, PA message, post-PA message, and number of trains.

Each variable independently associated with number of stair climbers modified the effect of the others. Examining the interaction between PODPs and gender, we found that the PODPs were less effective in the males than in the females with a factor of 0.93 (95% CI 0.92, 0.94). On the other hand, the rate of increase in number of male stair climbers as the number of trains increased was larger by a factor of 1.01 (95% CI 1.00, 1.01). 

## 4. Discussion

The use of stair-riser POPDs in this study was effective in increasing incidence of stair climbing in the experimental train station, compared to pre and post PODP periods. The multivariate negative binomial GEE model accounted for the control station and indicated a 48.5% increase in the likelihood in stair climbing during PODP intervention period and a 12% drop in stair climbing 2 weeks post-PODP. The expected count of stair climbers by combination by gender and number of trains per hour is shown in (Figure 2).

Our findings are consistent with conclusions provided in recent systematic reviews, which summarized the general effectiveness of PODPs [6,7]. In one review, it was reported that stair riser PODPs were effective in promoting use of stairs over escalators, but not elevators [6]. A recent economic analysis of various physical activity interventions found PODPs to be the most cost-effective strategy, reaching a large number of people with relatively low-cost efforts [19]. Although the amount of physical activity is less than the recommended 10 min bout [20], the volume may be significant when accumulated over the long run as found in one prospective study where by men had a 29% decrease in stroke risk associated with stair use [10]. A more recent study has demonstrated that even very short bouts of non-structured physical activity lasting between 32 s to 5 min can change the levels of blood lipids [11]. In addition, it has been demonstrated that incidental moderate to vigorous physical activity is positively associated with higher levels of CRF in inactive people [12]. At a population level, long term increases in energy expenditure and fitness level could generate significant health outcomes. 

Similar to our study, some studies [6,13] have also reported gender differences in response towards interventions used to promote stair climbing, with greater effects generally observed in females. Overall, our study indicated that the number of trains had a slight effect (6%) on the likelihood of stair climbing, while being male had a more substantial effect (22%). This may, in part, be explained by the physiological differences between males and females. Males may perceive stair climbing to be less challenging because they relatively tend to have greater lower body strength and higher cardiorespiratory fitness levels [13]. Upon further examination of the interaction of PODP’s with gender, we found that males appeared to be less receptive (7%) to PODPs than females and also appeared to be more likely to climb the stairs as related to an increase in the number of trains. This effect could be, in part, explained by psychosocial differences, whereby males were less receptive to the PODP messaging or simply less likely to wait in line for the escalator [14]. 

While use of PODPs significantly increased the number of stair climbers, the effect was lost after removal of intervention. The number of stair climbers went below baseline 2 weeks after PODPs were removed. In a similar study that demonstrated a residual increase in female stair climbers 2 weeks after removal of PODPs, intervention lasted 8 weeks while ours lasted only 4 weeks [14,15] This absence of residual effects in our study is possibly because the length of time for which intervention was in place was not sufficient to cultivate stair climbing habits in commuters. 

Despite the fact we were able to compare two identical station stairways with adjacent escalators, there were limitations to this investigation. We were only given permission to count the number of stair climbers, and were not able to capture escalator users. Consequently, we used number of train counts as a proxy for pedestrian flow and we verified with SMRT that overall pedestrian traffic of the respective stations was consistent during the period that the study was done. Markedly, during the intervention count period, there were fewer trains as compared to the pre-post periods but the absolute number of stair climbers was almost double. In addition, the control number of stair climbers over the duration of the study was not significantly different from period to period. Because we were not able to account for possible building design effects or other exit types across the experimental or control stations, using train count opposed to escalator users was a more practical approach for comparison opposed to accounting for every exit type in each station. We also were not able to factor for age and ethnicity because we were not given permission to interact with commuters. Some evidence exists to support the consistency of PODPs across building types. Lee *et al*. [21] found that using a standard size PODP placed at eye level directly adjacent to elevators and stairwells both increased and sustained stair use up to 9 months. Future PODP studies should also examine the influence of stair length and location on commuters’ decisions to use stairs *vs.* escalator. Such information would be valuable for architects who design MRT stations. 

## 5. Conclusions

In conclusion, our study is the first PODP intervention study that accounted for a control site, thereby providing evidence that PODPs are effective increasing stair climbing in the context of a multi-ethnic country such as Singapore. This knowledge adds to the growing body of evidence that using PODPs as a population health strategy to increase physical activity is effective across various cultural and ethnic settings. 
